# Sperm quality and testicular histopathology of Wistar albino male rats treated with hydroethanolic extract of *Cordia dichotoma* fruits 

**DOI:** 10.1080/13880209.2021.2008455

**Published:** 2022-02-09

**Authors:** Samah A. El-Newary, Mohamed S. Aly, Amal R. Abd El Hameed, Mohamed S. Kotp, Abdelghany A. Youssef, Naglaa A. Ali

**Affiliations:** a Medicinal and Aromatic Plants Research Department, National Research Centre, Dokki, Egypt; b Department of Animal Reproduction and Artificial Insemination, National Research Centre, Dokki, Egypt; c Hormones Department, National Research Centre, Dokki, Egypt

**Keywords:** Seminal quality, FSH and LH production, testosterone, histopathological studies

## Abstract

**Context:**

*Cordia dichotoma* Forst. (Boraginaceae) has potent pharmacological impact. Meanwhile, its effect on fertility is unclear.

**Objective:**

This study investigates the effect of *Cordia* fresh fruits hydroethanolic extract on fertility.

**Materials and methods:**

120 Wistar albino male rats were divided into four groups (*n* = 30). The first group was negative control, and the second, third, and fourth groups received 125, 250, and 500 mg extract/kg bodyweight for 56 days. After 56 days, *Cordia* force-feeding stopped, and all groups were kept under laboratory conditions for another month to study the recovering effect.

**Results:**

After day 56, extract at 500 mg/kg significantly reduced sperm total count, motility%, and alive%, to 47.60 ± 2.27 × 10^6^ sperm/mL, 43.33% ± 1.49, and 63.67% ± 1.19, respectively, abnormalities% increased considerably (26.67% ± 0.54), compared to the negative control. Also, significant depletion on follicle-stimulating hormone (2.66 ± 0.21 mIU/L), luteinizing hormone (1.07 ± 0.06 mIU/L), and testosterone (2.69 ± 0.13 nmol/L) level was recorded, compared to the negative control. *Cordia* negative effect showed on histopathological studies of testes, prostate, and seminal vesicles. Fortunately, these adverse effects of *Cordia* recovered remarkably after stopping administration for one month.

**Conclusions:**

*Cordia* antifertility effect may be due to its hypocholesterolemic effect, where cholesterol, the steroid cycle precursor, was significantly reduced. This study can be incorporated in clinical research after being repeated on another small experimental animal, their offspring, and one large experimental animal, then going to a clinical study that we plan to do in the future.

## Introduction

Fertility is a multi-factorial character; infertility is the diminished or absent capacity to produce viable offspring. Functional causes of infertility are secondary to the essential nutritional, hereditary, stress, and work factors (Abraham 2017). Male infertility is one of the most critical reproductive disorders strongly driven by environmental conditions (Mima et al. 2018). The defective sperm function has been identified as the most common cause of infertility (Adewoyin et al. 2017). Environmental exposures, including endocrine-disrupting chemicals and lifestyle exposures such as stress and feeding, can alter the epigenetic marks in the testicular germline and the spermatozoa (Cescon et al. 2020). Another critical factor in male fertility is sperm morphology. It is reported that abnormalities in sperm morphology reduce male reproductive potential (Ben Khelifa et al. 2014).

*Cordia dichotoma* Forst. (Boraginaceae) grows in Egypt and has been known as Mokhate trees since the Pharaoh's era (Tackholm 1974). The fruits are globose, yellowish-brown, pink or black, and pulpy. This fruit is divided into the pulpy part and one kernel seed (Nazim and Kakoti 2013). The fruits have been used to treat immunity defects, diabetes, liver diseases, fever, depression, and cooling. Also, fruits have been used as an emollient, laxative, astringent, expectorant, anthelmintic, purgative, diuretic, demulcent, female contraceptive, and cosmetic agent (Singh et al. 2010). *Cordia* fruits have many potent pharmacological impact as hypoglycaemic (Mishra and Garg 2010), antiulcer (Shah et al. 2011), anthelmintic and antimicrobial materials (Maisale et al. 2010), wound-healing agent (Kuppasta and Nayak 2006), hepatoprotective plant (Thirupathi et al. 2007), antioxidant (El-Newary et al. 2018), anti-inflammatory (Sharma et al. 2014), hypolipidemic (Sulieman and El-Newary 2014), and anticancer and antitumor (Ibrahim et al. 2019). Kuppast et al. (2010) demonstrated the aphrodisiac effect of *Cordia* fruits as an improvement in copulatory behaviour viz. sniffing, genital grooming, mounting frequencies, and the number of mating in albino rats. On the contrary, Sharma et al. (2015) reported that *Cordia* leaves are traditionally used to produce sterility among the tribal women through their abortifacient activity. They proved the antifertility effect of *Cordia* leaves in female rats. Because the tree is present in the field, farm animals can eat it. Based on the previous interpretation, *Cordia* fruits were subjected to this study to determine if they are a fertility stimulator or fertility inhibitor. The current study was planned to describe the effect of *C. dichotoma* fruits hydroethanolic extract on fertility using Wistar male albino rats.

## Materials and methods

### Chemicals

Folin-Ciocalteu reagent and authentic samples of gallic acid and quercetin were purchased from Sigma- Aldrich (St. Louis, MO, USA). All other chemicals were of analytical grade. Kits for spectrophotometric analysis were obtained from Bio Diagnostic (Giza, Egypt). ELISA kit for insulin was purchased from Epitope Diagnostic Inc Company, San Diego, USA. ELISA kits for follicle-stimulating hormone (FSH) and Luteinizing hormone (LH) were purchased from AB diagnostic system Gmbh, Berlin, Germany. ELISA kit for testosterone (TS) hormone was purchased from XEMA Co. Ltd, Moscow, Russia.

### Collection of *C. dichotoma* fruit and preparation of the extract

*C. dichotoma* fruits were collected from *Cordia* trees in Sharkia Governorate, Egypt, during August 2018. The plant was identified by the Department of Plant Taxonomy at the Ministry of culture and Orman Botanical Garden, Giza, Egypt. A voucher specimen (no. 1-18-5) was deposited in the herbarium of Mazhar Shehab Botanical Garden, Giza, Egypt. Washed fruits (1 kg) were crushed using a blender (Toshiba) and were exhaustively extracted with 70% ethyl alcohol (3 L) by shacked soaking several times (4 times) during 1 month at room temperature. The filtrate was collected every 1 week, evaporated by a rotary evaporator at 40 °C, and then lyophilised. The remaining residue (200 *g*) was kept under −20 °C.

### Determination of the chemical composition of *Cordia* extract

#### Determination of total phenolic content

The total phenol content was determined according to Singleton et al. (1999) and was expressed as mg gallic acid/g of extract. Extract (1 mL), distilled water (9 mL), and Folin-Ciocalteu phenol reagent (1 mL) were shaken in a volumetric flask (25 mL). After 5 min, 7% NaCO_3_ solution (10 mL) was added, and volume was completed to 25 mL with distilled water. Gallic acid (standard solution) (20, 40, 40, 60, 80, and 100 μg/mL) was prepared with the same procedure. The reaction was incubated (90 min at room temperature) and read at 550 nm with an Ultraviolet (UV)/Visible spectrophotometer against blank.

#### Determination of total flavonoid content

Total flavonoid content was measured according to Lin and Tang (2007) and was expressed as mg quercetin/g of extract. Extract (1 mL), distilled water (4 mL), and 5% NaNO_2_ (0.30 mL) were taken in a 10 mL volumetric flask. 10% AlCl_3_ (0.3 mL) was added after 5 min. NaOH (2 mL 1 M) was added after 5 min and diluted to 10 mL with distilled water. Quercetin (standard solution) (20, 40, 60, 80 and 100 μg/mL) was prepared with the same procedure. The reaction was read at 510 nm against blank.

#### Determination of crude alkaloids

The gravimetric method of Onwuka (2006) was used. First, 5 *g* of extract was extracted in 50 mL of 10% acetic acid solution in absolute ethanol by shaken for 4 h. Then, concentrated NH_4_OH was added drop by drop to precipitate the alkaloids. The precipitate (in weighted filter paper) was filtrated off and washed with 1% NH_4_OH solution. The filter paper with alkaloids was dried at 60 °C for 60 min and reweighted. By weight difference, the weight of alkaloids was determined.

#### HPLC analysis

The extracts were analysed on Shimadzu Class-VPV 5.03 (Kyoto, Japan) equipped with Shimadzu UV-Vis detector (SPD-10Avp) at 330 nm, LC-16ADVP binary pump, DCou-14 A degasser, and Phenomenex RP-18 (UK; 250 **×** 4.00 mm, 5 µ) column according to Kim et al. (2006). The solvent gradient used in this study was formed through a solvent (A methanol and B 0.03% phosphoric acid in water). The linear gradient was used for chromatographic separation: 0 min, 70% B; 6 min, 55% B; 20 min, 45% B. The run time was 30 min. The injection volume was 5 µL. The solvent flow rate was maintained at 1.0 mL/min. All analyses were carried out at 30 °C. The spectra were recorded at 330 nm. The method of the external standard was used for quantification.

### Assay of acute oral toxicity (LD_50_)

Acute toxicity of the *Cordia* fruits extract was performed according to per OECD guideline 425 (OECD 2008) for acute oral toxicity -Up-and-Down- Procedure (UDP). The dosing pattern started from 1000 to 10,000 mg/kg body weight, with a rate of 1000 mg/kg body weight. Mice force-fed the extract by gastric tube (5 mice), and control mice received saline only. All groups were kept under observation and were checked out for any changes and mortality through 48 h. Alive animals were observed for 14 days. Using mortality number in each concentration during the first 48 h and BioStat program (BioStat 2009 Build 5.8.4.3 © 2021 analyst Soft Inc., VA, USA), the extract dose killed 50% of the animals (LD_50_) was estimated at 10,000 mg/kg.

### The antifertility effect of *Cordia* extract

#### Ethical, animals and accommodations

The experiment was conducted in the animal house at the National Research Centre, Dokki, Giza, Egypt. Permission was obtained from the Ethics Committee of the National Research Centre under registration No. 19/093. Adult male Wistar albino rats (120 rats) ranging from 150 to 170 g in weight were obtained from the Central Animal House at the National Research Centre, Giza, Egypt. Animals were maintained in plastic cages under laboratory conditions (20–25 °C, 55–65% humidity, and 10–12 h light/dark cycle). Water and food were available *ad libitum* over three months.

#### Experimental design

The extract three doses of 500, 250, 125 mg/kg body weight were dissolved in normal saline and were administrated by gastric tube. Experimental animals were adapted for 1 week under laboratory conditions. The 120-rats were divided into four groups, each group 30 rats. The first group was the negative control group; the rats were force-fed normal saline for day 56. The second, third, and fourth groups were e force-fed the extract as 125, 250, 500 mg/kg body weight, respectively, for 56 days. After day 56, *Cordia* force-feeding stopped, and the four groups were kept under laboratory conditions for another month to study the recovery effect.

After 28-days, day-56, and day-90, the experimental period, ten rats from each group were fasted and maintained with tap water overnight. Rats were anaesthetised by injecting ketamine and xylazine (87 and 13 mg/kg, respectively, dissolved in normal saline; and each rat received a 0.2 mL/100 *g* body weight) (Van Pelt 1977). The blood samples were collected from the retro-orbital plexus before animals were sacrificed then organs were dissected. Organs were washed and weighed freshly for chronic toxicity evaluation. Sera were collected after blood centrifugation at 3500 *g* for 10 min using Sigma Laborzentrifugen (Osterode am Harz, Germany). Testes, prostate, and seminal vesicles were kept in 10% formalin for histopathological examination.

#### Blood glucose level following up

Every month, rats fasted overnight, and blood samples were collected from each rat's tip of tail veins (at 9 a.m.). Glucose concentration was estimated immediately, using Gluco Star Test Strip (Taidoc Technology Corp., New Taipei, Taiwan).

### Semen analysis

Immediately after the rats were sacrificed, epididymis' contents were obtained by cutting the caudal epididymis, and sperm samples were collected using the method described by Yokoi et al. (2003). The sperm suspension was carefully mixed with an equal volume of *eosin–nigrosine* stain to evaluate sperm viability, where eosin-stained dead sperm pink and live sperm remained unstained (Wyrobek et al. 1983).

#### Biochemical assessment

Biochemical analyses were determined in serum spectrophotometrically. Liver function as total protein (Henry 1964), albumin (Doumas et al. 1971), aspartate aminotransferase (AST), and alanine aminotransferase (ALT) (Reitman and Frankel 1957) were determined. Kidney function as urea (Tabacco et al. 1979), uric acid (Gochman and Schmitz 1971), and creatinine (Faulkner and King 1976) were estimated. Lipid profile was evaluated, including; total cholesterol (TC) (Allain et al. 1974), high-density lipoprotein cholesterol (HDL-C) (Naito and Kaplan 1984), and triglycerides (TG) (Fossati and Prencipe 1982). In addition, low-density lipoprotein cholesterol (LDL-C) (Naito and Kaplan 1984) and very-low-density lipoprotein cholesterol (VLDL-C) (Friedewald et al. 1972) were calculated.

Antioxidant biomarkers, reduced glutathione (GSH), glutathione reductase (GR), glutathione-*S*-transferase (GST), glutathione peroxidase (GPx), and catalase (CAT) were determined according to Griffith (1980), Goldberg and Spooner (1983), Paglia and Valentine (1967), Habig et al. (1974), Beers and Sizer (1952), respectively. Oxidative stress biomarkers, malondialdehyde (MDA), and hydrogen peroxide (H_2_O_2_) were estimated according to Ohkawa et al. (1979), Chance and Maehly (1955), respectively. According to the manufactured instruction, an enzyme-linked immunosorbent assay (ELISA) was performed for the quantitative measurement of insulin, FSH, LH, and TS levels in serum samples.

#### Histopathological analysis

Histopathological samples (testes, prostate, and seminal vesicle) were prepared and stained with Haematoxylin and Eosin **(**Drury and Wallington 1980) for light microscope examination (Olympus CX 41, Japan).

### Statistical analysis

Data were presented as mean ± SE (standard error), *n* = 10. A one-way ANOVA test was performed to compare different groups, followed by Duncan's *post hoc* test, and all comparisons were significant when *p* ≤ 0.05 using software COSTAT (version 6.400, Cohort Software, Birmingham, UK).

### Results

#### Polyphenol, flavonoid, and alkaloid content of *Cordia* extract

This study determined the total phenols, flavonoids, and alkaloids of *Cordia* fresh fruit and hydroethanolic extract of fresh fruits (70%). The total phenolic content in fresh fruits and *Cordia* extract was 2.95 ± 0.13 and 3.01 ± 0.18 mg gallic acid/g extract. The total flavonoid content of the fresh fruits and extract was evaluated as 3.87 ± 0.17 and 4.26 ± 0.14 mg quercetin/g extract, respectively. Total alkaloids of *Cordia* fresh fruit and extract were 6.50 ± 0.25 and 56.95 ± 0.75 mg total alkaloids/g.

**Figure 1.**
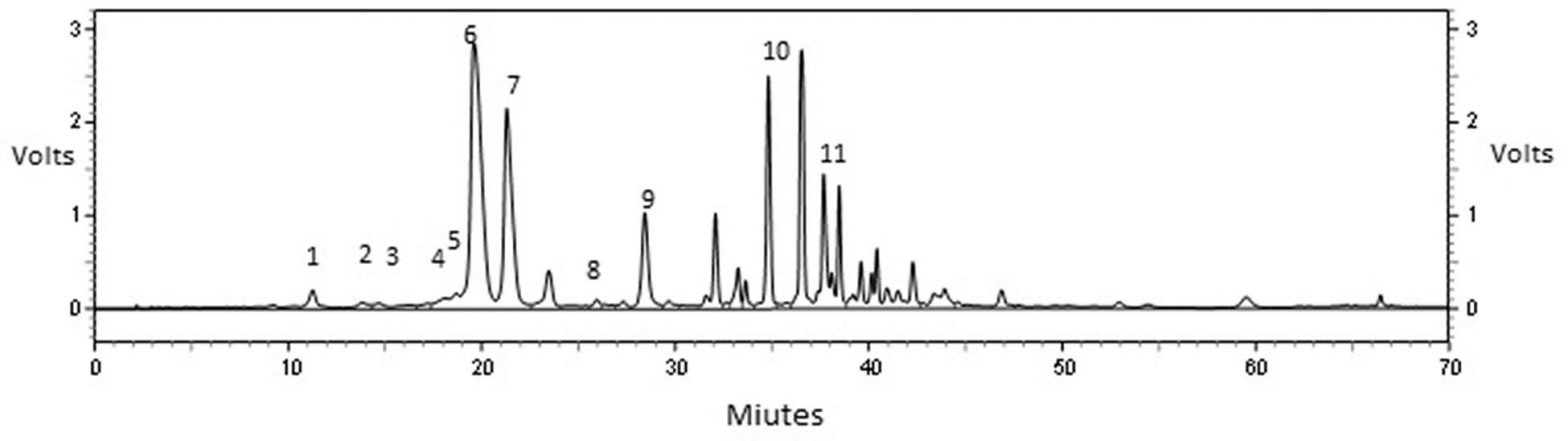
HPLC analysis of *Cordia dichotoma* fresh fruits hydroethanolic extract. Where 1 means gallic acid, 2 means chlorogenic acid, 3 means caffeic acid, 4 means rutin, 5 means quercetin, 6 rosmarinic acid, 7 means kaempferol, 8 means *p*-qumaric, 9 means syringic acid, 10 means vanillic acid, and11 means salicylic acid.

HPLC analysis showed eight phenolic acids in the extract, including gallic acid (2.64%), chlorogenic acid (0.12%), caffeic acid (1.35%), rosemarinic acid (35.78%), *p*-qumaric (0.37%), syringic (4.82%), vanillic (6.19%), and salicylic acid (3.28%). In addition to three flavonoids, including rutin (7.92%), quercetin (5.19%), and kaempferol (10.64%) (Figure 1).

### Acute toxicity

Oral administration of a single dose of graded concentrations of *Cordia* fruits extract (1–7 g/kg body weight) to albino mice groups did not produce any mortality or dangerous changes during the first 48 h. After the first 48 h, no mortality or detrimental changes were observed during the following 14 days with these concentrations, compared to the negative control. The mortality started with concentration at 8 g/kg/day until 10 g/kg/day. The concentration that killed 50% of animals during the first 48 h was estimated at 10 g/kg/day.

### The effect of *Cordia* extract on safety evaluation

#### Effect on monthly body weight gain

**Figure 2.**
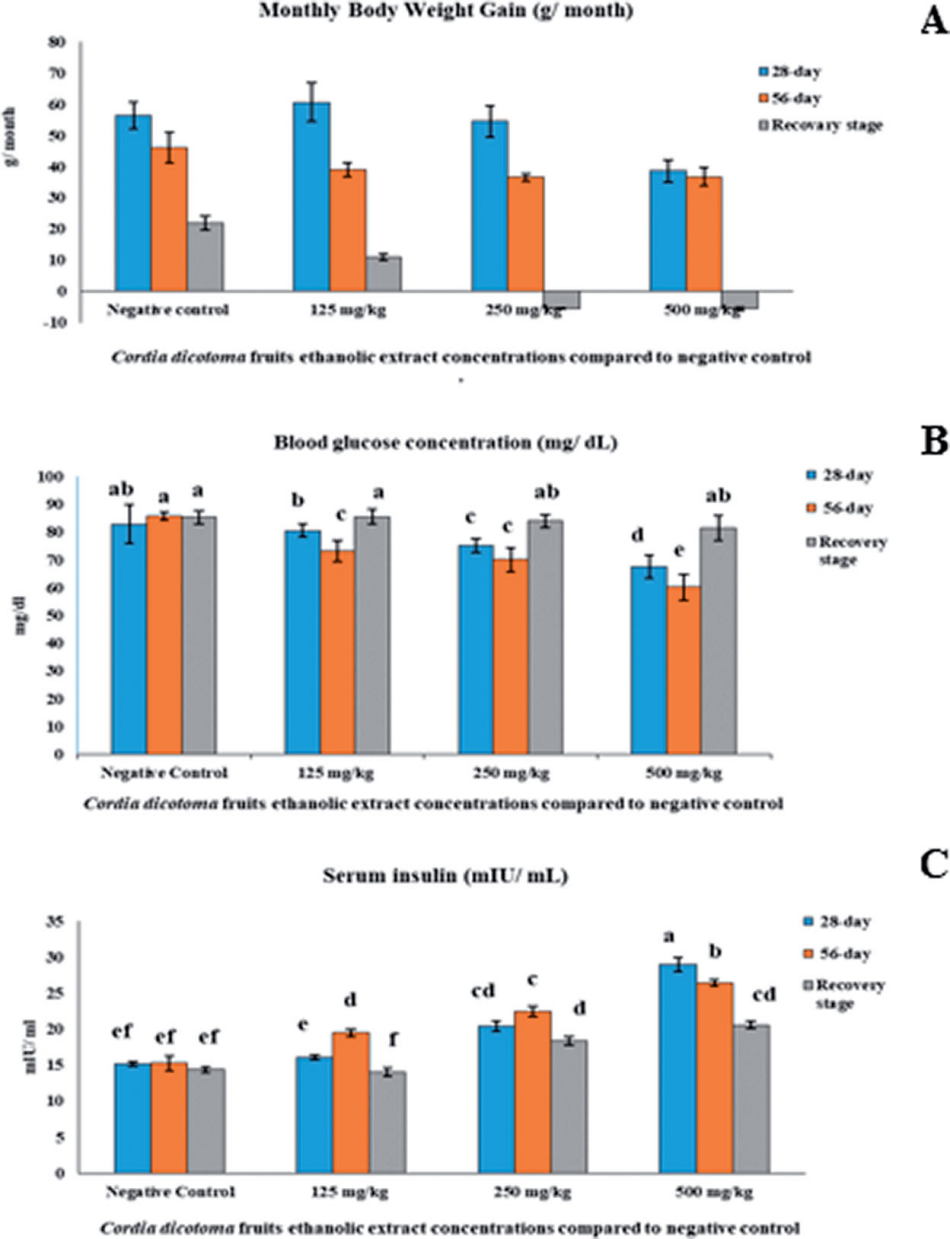
Monthly body weight gain (A), blood glucose concentration (B), and serum insulin concentration (C) of Wistar male rats treated with *Cordia dichotoma* fruits hydroethanolic extract compared to the negative control. Data presented are mean ± SE.

Compared to the negative control, Cordia extract treatments significantly affected monthly body weight gain (MBWG) (*p* ≤ 0.05). In addition, indirect relation between *Cordia* concentration and MBWG was noticed, where MBWG decreased when *Cordia* doses increased (Figure 2(A)).

After 28 days, the bodyweight of the high dose group significantly (*p* ≤ 0.05) decreased compared with the control group. After day 56, the bodyweight of both medium and high doses significantly (*p* ≤ 0.05) decreased than the control group, and the three groups showed a decrease in recovery month compared with the control.

#### Effect on the liver and renal functions

Data presented in Table 1 showed that *Cordia* treatments had no significant effect on liver functions, including total protein and its two-fraction albumin and globulin levels and AST and ALT activities of treated rats along the experimental period negative control (*p* ≤ 0.05). Table 1 shows that renal functions biomarkers, creatinine, uric acid, and urea were significantly (*p* ≤ 0.05) reduced as a response to *Cordia* administration compared to the corresponding values of the negative control (*p* ≤ 0.05).

**Table 1.** Serum liver and kidney functions of male rats treated with *Cordia dichotoma* fruits ethanolic extract compared to the negative control.

Parameters	Serum liver functions					Serum kidney functions		
Groups	TP (g/dl)	Albumin (g/dl)	Globulin |(g/dl)	AST (U/L)	ALT (U/L)	Creatinine (mg/dL)	Uric acid |(mg/dL)	Urea |(mg/ dL)
After administration for 28-day								
Negative control	7.28 ± 0.20^a^	4.82 ± 0.24^ab^	2.46 ± 0.32^a^	45.31 ± 1.55^a^	23.56 ± 0.87^a^	1.39 ± 0.02^a^	4.02 ± 0.02^a^	25.21 ± 0.07^a^
Cordia 125 mg/kg	7.30 ± 0.13^a^	4.95 ± 0.31^ab^	2.35 ± 0.38^a^	44.85 ± 1.04^a^	23.48 ± 1.27^a^	1.06 ± 0.03^b^	3.34 ± 0.09^b^	25.23 ± 0.13^a^
Cordia 250 mg/kg	7.37 ± 0.51^a^	5.04 ± 0.36^ab^	2.49 ± 0.50^a^	45.62 ± 2.20^a^	23.69 ± 1.26^a^	0.92 ± 0.02^c^	2.22 ± 0.01^d^	22.80 ± 0.18^b^
Cordia 500 mg/kg	7.31 ± 0.53^a^	4.99 ± 0.40^ab^	2.31 ± 0.24^a^	45.96 ± 1.26^a^	22.51 ± 1.28^a^	0.78 ± 0.02^d^	1.51 ± 0.02^g^	22.92 ± 0.16^b^
After administration for 56-day								
Negative control	7.40 ± 0.15^a^	4.92 ± 0.19^ab^	2.48 ± 0.25^a^	45.89 ± 1.18^a^	22.79 ± 1.08^a^	1.04 ± 0.01^b^	3.22 ± 0.04^b^	24.50 ± 0.06^a^
Cordia 125 mg/kg	7.56 ± 0.43^a^	5.19 ± 0.52^a^	2.36 ± 0.32^a^	45.97 ± 0.84^a^	23.00 ± 0.99^a^	0.70 ± 0.02^e^	2.46 ± 0.09^c^	19.14 ± 0.31^d^
Cordia 250 mg/kg	7.42 ± 0.42^a^	4.84 ± 0.44^abc^	2.59 ± 0.05^a^	44.80 ± 0.99^a^	22.13 ± 1.11^a^	0.68 ± 0.03^e^	2.02 ± 0.04^ef^	21.43 ± 0.34^c^
Cordia 500 mg/kg	7.49 ± 0.20^a^	5.24 ± 0.15^a^	2.45 ± 0.17^a^	44.90 ± 1.38^a^	22.61 ± 1.32^a^	0.55 ± 0.03^f^	1.37 ± 0.06^g^	21.88 ± 0.31^d^
Recovery stage								
Negative control	7.19 ± 0.41^a^	4.70 ± 0.15^b^	2.49 ± 0.44^a^	44.96 ± 0.66^a^	23.95 ± 10.66^a^	1.03 ± 0.03^b^	2.11 ± 0.04^de^	21.00 ± 0.40^c^
Cordia 125 mg/kg	7.32 ± 0.49^a^	4.94 ± 0.47^ab^	2.38 ± 0.23^a^	44.28 ± 0.56^a^	23.86 ± 159^a^	0.97 ± 0.02^bc^	1.99 ± 0.04^ef^	19.68 ± 0.24^d^
Cordia 250 mg/kg	7.47 ± 0.37^a^	5.06 ± 0.39^ab^	2.45 ± 0.26^a^	45.58 ± 0.40^a^	23.39 ± 1.41^a^	0.97 ± 0.03^bc^	1.90 ± 0.04^f^	14.53 ± 0.25^e^
Cordia 500 mg/kg	7.44 ± 0.33^a^	5.02 ± 0.24^ab^	2.47 ± 0.24^a^	44.48 ± 0.72^a^	23.11 ± 1.28^a^	1.04 ± 0.02^b^	1.22 ± 0.05^h^	14.50 ± 0.43^e^

Data presented as mean ± SE. Data were analysed using ANOVA one-way followed with *post hoc* for multiple comparisons and *P < 0.05*. The means followed by the same letter in each column are not significantly different from each other at 5% probability level (Duncan’s multiple range test). TP: total protein, AST: Aspartate aminotransferase; ALT: alanine aminotransferase.

Creatinine, uric acid, and urea levels of *Cordia*-treated groups significantly declined after administration of the extract for 28-days, and this reduction continued for 56 days, compared to the negative control (*p* ≤ 0.05). The same observation was recorded at the recovery stage.

#### The effect of *Cordia* extract on blood glucose and serum insulin

*Cordia* extract showed a hypoglycaemic effect in a dependent manner that represented through a significant (*p* ≤ 0.05) reduction in blood glucose levels concurrent with a significant (*p* ≤ 0.05) rise in serum insulin, compared to the negative control values (Figure 2(B)). Blood glucose levels of rats treated with *Cordia* extract at all doses were significantly diminished from month to month to reach the minimum level at day 56, and then it was non-significantly increased at the recovery stage.

On the contrary, serum insulin of *Cordia*-treated animals significantly raised until the end of the experiment. (Figure 2(B)). The lowest blood glucose level during the experiment was recorded at day 56 with the highest dose of *Cordia* extract (60.20 ± 4.62 mg/dL) in comparison with the negative control level at the same time (85.75 ± 1.39 mg/dL). At the recovery stage, blood glucose of *Cordia*-groups was significantly increased compared to previous values but was still lower than that of the negative control.

Serum insulin levels of *Cordia*-treated groups were significantly elevated after administering the extract for 28 and 56 days, compared to the negative control (Figure 2(C)). After 28-day, the highest *Cordia* dose recorded the highest insulin level (29.04 ± 0.95 mIU/mL) during the experimental period. *Cordia*-groups' insulin levels were significantly higher than that of the negative control at the recovery stage.

#### The effect of *Cordia* extract on serum lipid profile

The extract exhibited a hypolipidemic impact on the treatment and recovery stages compared to the negative control (Table 2). The hypolipidemic result of *Cordia* was in a dependent manner. TC, TG, VLDL-C, and LDL-C levels of *Cordia*-treated groups significantly declined after administering the extract for 28 and 56 days (*p* ≤ 0.05). The hypolipidemic effect of *Cordia* after administration for 56 days was higher than its effect after 28-day. After day 56, the highest dose of *Cordia* produced the lowest values of TC, TG, and VLDL-C (75.13 ± 0.31, 107.85 ± 2.12 and 21.57 ± 1.44, 2.70 ± 0.49 mg/dL, respectively) during the experimental period. At the recovery stage, TC, TG, VLDL-C, and LDL-C levels of *Cordia*-treated groups significantly elevated more than recorded at 28 and 56 days. However, they were still lower than the negative control at the same stage (*p* ≤ 0.05).

**Table 2.** Serum lipid profile of male rats treated with *Cordia dichotoma* fruits ethanolic extract compared to the negative control.

	Parameters					
Groups	TC (mg/dL)	TG (mg/dL)	VLDL-C (mg/dL)	HDL-C (mg/dL)	LDL-C (mg/dL)	Risk ratio RR (%)
After administration for 28-day						
Negative control	98.66 ± 0.77^b^	149.68 ± 1.47^b^	29.94 ± 0.88^b^	40.34 ± 0.48^f^	28.38 ± 0.44^ab^	0.71 ± 0.05^a^
Cordia 125 mg/kg	95.56 ± 2.54^b^	138.90 ± 2.27^c^	27.78 ± 1.33^c^	43.10 ± 0.72^e^	24.68 ± 2.97^abc^	0.58 ± 0.03^abc^
Cordia 250 mg/kg	87.10 ± 1.35^c^	139.36 ± 1.23^c^	27.87 ± 0.74^c^	47.38 ± 0.64^bcd^	11.85 ± 1.34^de^	0.25 ± 0.01^ef^
Cordia 500 mg/kg	78.72 ± 0.94^d^	111.36 ± 2.30^e^	22.27 ± 1.52^e^	49.43 ± 0.39^ab^	7.01 ± 0.90^ef^	0.14 ± 0.008^f^
After administration for 56-day						
Negative control	105.86 ± 2.94^a^	156.42 ± 2.96^ab^	31.28 ± 1.81^ab^	44.88 ± 0.89^de^	29.70 ± 1.99^a^	0.68 ± 0.06^a^
Cordia 125 mg/kg	88.20 ± 1.96^c^	138.07 ± 6.80^c^	27.61 ± 1.36^c^	44.00 ± 0.53^e^	16.58 ± 1.97^cd^	0.38 ± 0.01^de^
Cordia 250 mg/kg	78.97 ± 1.59^d^	124.53 ± 3.78^d^	24.91 ± 2.38^d^	47.27 ± 1.18^bcd^	6.79 ± 1.55^ef^	0.15 ± 0.01^f^
Cordia 500 mg/kg	75.13 ± 0.31^d^	107.85 ± 2.12^e^	21.57 ± 1.44^e^	50.87 ± 1.11^a^	2.70 ± 0.49^f^	0.06 ± 0.004^f^
Recovery stage						
Negative control	109.85 ± 2.55^a^	160.45 ± 1.95^a^	32.09 ± 1.17^a^	48.06 ± 0.49^bc^	29.70 ± 2.70^a^	0.62 ± 0.03^ab^
Cordia 125 mg/kg	97.94 ± 1.47^b^	140.25 ± 2.51^c^	28.05 ± 1.58^c^	46.38 ± 0.42^cd^	23.51 ± 2.18^abc^	0.51 ± 0.02^abcd^
Cordia 250 mg/kg	97.16 ± 2.06^b^	132.00 ± 2.49^c^	26.40 ± 1.50^c^	49.40 ± 0.36^ab^	21.37 ± 2.36^abc^	0.43 ± 0.01^bcd^
Cordia 500 mg/kg	93.32 ± 2.48^b^	115.80 ± 3.22^e^	23.16 ± 1.96^e^	49.52 ± 0.47^ab^	20.64 ± 0.75^bc^	0.42 ± 0.013^cde^

Data presented as mean ± SE. Data were analysed using ANOVA one-way followed with *post hoc* for multiple comparisons and *P < 0.05*. The means followed by the same letter in each column are not significantly different from each other at 5% probability level (Duncan’s multiple range test). TC: total cholesterol; TG: triglycerides; VLDL-C: very low-density lipoprotein; LDL-C: low-density lipoprotein; HDL-C: high-density lipoprotein. VLDL-C = TG/5, LDL-C = TC- (HDL-C + VLDL-C), and risk ratio = LDL-C/ HDL-C.

On the contrary, HDL-C levels of *Cordia*-treated groups significantly magnified after administration *Cordia* extract for 28 and 56 days, compared to each negative control. After day 56, the highest dose of *Cordia* produced the highest HDL-C level (50.87 ± 1.11 mg/dL) during the experimental period. The HDL-C level did not change significantly at the recovery stage compared to the negative control at the same stage.

#### The effect of *Cordia* extract on antioxidant biomarkers

*Cordia* treatments had significant antioxidant properties, which parallelly increased by *Cordia* doses increased during treatment and recovery stages compared to the negative control (*p* ≤ 0.05) (Table 3). Administration of *Cordia* for 28 and 56 days significantly raised antioxidant biomarkers levels, including GSH concentration, GR, GST, and GPx activities of *Cordia*-treated groups serum compared to the negative control (*p* ≤ 0.05). The ameliorative effect of *Cordia* extract on antioxidant biomarkers prolonged the recovery stage. The highest GSH concentration, GR, GST, and GPx activities were recorded at the recovery stage (Table 3). CAT activity did not significantly change in all *Cordia*-treated groups at the treatment or recovery stage than the negative control. Force-feeding *Cordia* doses significantly minified MDA and H_2_O_2_ levels, the oxidative stress biomarkers at treatment and recovery stages, compared to the negative control (*p* ≤ 0.05) (Table 3).

**Table 3.** Antioxidant biomarkers and oxidative stress biomarkers of male rats treated with *Cordia dichotoma* fruits ethanolic extract compared to a negative control.

Parameters	Antioxidant biomarkers					Oxidative stress biomarkers	
Groups	GSH (mg/dl)	GR (μmol/mg protein/min)	GST (μmol/mg protein/min)	GPx (μmol/mg protein/min)	CAT (μmol/mg protein/min)	MDA (nmol/mL)	H_2_O_2_ mmol/L.
After administration for 28-day							
Negative control	1.57 ± 0.04^f^	1.95 ± 0.05^f^	1.31 ± 0.04^f^	0.62 ± 0.02^g^	5.65 ± 0.12^a^	11.39 ± 0.16^a^	2.86 ± 0.06^bc^
Cordia 125 mg/kg	1.70 ± 0.04^f^	2.11 ± 0.05^f^	1.42 ± 0.03^f^	1.40 ± 0.02^d^	5.89 ± 0.05^a^	11.55 ± 0.02^a^	2.42 ± 0.10^cd^
Cordia 250 mg/kg	2.35 ± 0.09^de^	2.91 ± 0.11^de^	1.96 ± 0.08^de^	1.54 ± 0.03^c^	5.84 ± 0.10^a^	8.72 ± 0.14^b^	1.83 ± 0.11^e^
Cordia 500 mg/kg	2.87 ± 0.03^c^	3.57 ± 0.03^c^	2.40 ± 0.02^c^	2.14 ± 0.03^a^	6.09 ± 0.12^a^	5.43 ± 0.09^f^	1.61 ± 0.06^e^
After administration for 56-day							
Negative control	1.74 ± 0.06^f^	2.16 ± 0.08^f^	1.45 ± 0.05^f^	0.66 ± 0.02^f^	5.66 ± 0.38^a^	7.49 ± 0.09^d^	2.9 ± 0.03^b^
Cordia 125 mg/kg	2.12 ± 0.07^e^	2.63 ± 0.09^e^	1.77 ± 0.06^e^	1.38 ± 0.01^d^	5.95 ± 0.03^a^	7.43 ± 0.09^d^	2.72 ± 0.11^bc^
Cordia 250 mg/kg	2.51 ± 0.09^d^	3.11 ± 0.11^d^	2.08 ± 0.07^d^	1.82 ± 0.05^b^	6.05 ± 0.18^a^	4.52 ± 0.09^h^	2.56 ± 0.08^bcd^
Cordia 500 mg/kg	3.19 ± 0.06^b^	3.95 ± 0.08^b^	2.66 ± 0.05^b^	2.27 ± 0.11^a^	6.32 ± 0.08^a^	5.01 ± 0.07^g^	2.20 ± 0.08^d^
Recovery stage							
Negative control	2.19 ± 0.05^e^	2.72 ± 0.07^e^	1.21 ± 0.04^e^	0.67 ± 0.04^f^	6.01 ± 0.26^a^	7.88 ± 0.09^c^	4.55 ± 0.07^a^
Cordia 125 mg/kg	2.24 ± 0.07^e^	2.79 ± 0.08^e^	1.87 ± 0.06^e^	1.16 ± 0.04^e^	6.00 ± 0.14^a^	6.81 ± 0.06^e^	4.47 ± 0.03^a^
Cordia 250 mg/kg	2.57 ± 0.06^d^	3.18 ± 0.08^d^	2.13 ± 0.05^d^	1.62 ± 0.03^c^	6.07 ± 0.24^a^	3.63 ± 0.06^j^	3.07 ± 0.33^b^
Cordia 500 mg/kg	4.025 ± 0.07^a^	5.00 ± 0.08^a^	3.35 ± 0.06^a^	2.213 ± 0.11^a^	6.48 ± 0.20^a^	3.06 ± 0.05^j^	2.70 ± 0.12^bc^

Data presented as mean ± SE. Data were analysed using ANOVA one-way followed with *post hoc* for multiple comparisons and *P < 0.05*. The means followed by the same letter in each column are not significantly different from each other at the 5% probability level (Duncan’s multiple range test). GSH: glutathione L reduced; GR: glutathione reductase; GST: glutathione -S- transferase; GPx: glutathione peroxidase; CAT: catalase; MDA: malondialdehyde; H_2_O_2_: hydrogen peroxide.

### Effect of *Cordia* extract on male fertility

#### The impact on sex hormones

Data in Table 4 show that *Cordia* treatments recorded a significant (*p* ≤ 0.05) effect on FSH, LH, and TS production of all *Cordia*-treated groups, compared to the negative control. The general trend was observed, *Cordia* significantly decreased these hormones during the treatment stage and significantly increased them during the recovery stage.

**Table 4.** Serum hormone concentrations and seminal quality of male rats treated with *Cordia dichotoma* fruits ethanolic extract compared to the negative control.

Parameters	Serum hormone concentrations			Seminal quality			
Groups	FSH (mIU/ L)	LH (mIU/ L)	TS (nmol/ L)	Total count ×10^6^	Motility %	Life %	Abnormality %
After administration for 28-day							
Negative control	3.80 ± 0.33^d^	1.80 ± 0.09^e^	3.88 ± 0.38^a^	69.83 ± 2.78^cd^	53.33 ± 3.95^bc^	72.00 ± 2.37^ef^	19.33 ± 0.91^cd^
Cordia 125 mg/kg	3.60 ± 0.07^e^	1.89 ± 0.05^de^	3.84 ± 0.04^ab^	70.58 ± 4.67^cd^	53.33 ± 1.75^bc^	84.67 ± 1.49^ab^	18.00 ± 0.68^d^
Cordia 250 mg/kg	2.17 ± 0.01^h^	1.52 ± 0.06^f^	2.26 ± 0.13^f^	64.00 ± 1.33^ef^	50.00 ± 2.58^bc^	78.33 ± 1.81^cd^	27.33 ± 0.79^a^
Cordia 500 mg/kg	1.59 ± 0.13^i^	1.29 ± 0.16^g^	3.82 ± 0.11^ab^	55.04 ± 3.64^g^	40.00 ± 001^e^	66.33 ± 0.39^g^	23.00 ± 0.77^b^
After administration for 56-day							
Negative control	3.88 ± 0.14^d^	1.83 ± 0.03^e^	3.58 ± 0.22^b^	72.42 ± 5.75^bc^	66.67 ± 1.49^a^	82.00 ± 1.13^bc^	17.67 ± 0.91^de^
Cordia 125 mg/kg	3.97 ± 0.41^d^	1.97 ± 0.16^d^	3.33 ± 0.29^c^	79.50 ± 5.67^a^	57.67 ± 2.49^b^	76.00 ± 1.03^de^	21.00 ± 0.26^c^
Cordia 250 mg/kg	3.01 ± 0.03^f^	1.04 ± 0.02^h^	2.68 ± 0.24^e^	61.50 ± 3.62^f^	60.00 ± 2.58^ab^	81.00 ± 0.93^bc^	20.33 ± 0.15^c^
Cordia 500 mg/kg	2.66 ± 0.21^g^	1.07 ± 0.06^h^	2.69 ± 0.13^e^	47.60 ± 2.27^h^	43.33 ± 1.49^d^	63.67 ± 1.19^g^	26.67 ± 0.54^a^
Recovery stage							
Negative control	3.51 ± 0.30^e^	2.00 ± 0.15^d^	3.12 ± 0.26^d^	66.00 ± 6.93^def^	52.00 ± 2.49^bc^	67.20 ± 0.83^g^	19.60 ± 0.50^cd^
Cordia 125 mg/kg	4.24 ± 0.09^c^	2.45 ± 0.01^c^	3.59 ± 0.28^b^	67.50 ± 3.67^cde^	50.00 ± 1.29^bc^	71.33 ± 1.29^f^	25.33 ± 0.39^a^
Cordia 250 mg/kg	4.72 ± 0.07^b^	2.58 ± 0.14^b^	3.84 ± 0.08^ab^	68.68 ± 3.36^cde^	49.50 ± 0.01^cd^	72.67 ± 1.04^ef^	26.00 ± 0.45^a^
Cordia 500 mg/kg	5.33 ± 0.17^a^	2.76 ± 0.24^a^	4.05 ± 0.18^a^	76.20 ± 4.64^ab^	65.00 ± 1.29^a^	87.00 ± 0.45^a^	17.33 ± 0.15^d^

Data were presented as mean ± SE. Data were analysed using ANOVA one-way followed with *post hoc* for multiple comparisons and *P* < 0.05. The means followed by the same letter in each column are not significantly different from each other at 5% probability level (Duncan’s multiple range test).

Life% = (number of live sperms/ total sperms count) × 100

Abnormality% = (number of deformed sperms/ total sperms count) × 100.

Motility% = (number of motile sperms/ total sperms count) × 100.

*Cordia* treatment showed a negative effect on FSH and LH concentration, where gonadotropic cells produced FSH and LH less than negative control after 28-day (*p* ≤ 0.05), and the highest dose of *Cordia* caused the highest reduction in FSH (1.59 ± 0.13 and 1.29 ± 0.16 mIU/L, respectively), in comparison with the negative control (3.80 ± 0.33 and 1.80 ± 0.09 mIU/L, respectively). Ongoing *Cordia* administration (at medium and high doses) for 56 days showed the same trend and decreased FSH and LH levels significantly. The highest dose of *Cordia* recorded the lowest FSH and LH concentration (2.66 ± 0.2 and 1.07 ± 0.06 mIU/L, respectively), in comparison with the negative control (3.88 ± 0.14 and 1.83 ± 0.03 mIU/L, respectively). Thus, although *Cordia* caused a significant decrease in the FSH concentration after administration for 56 days, compared with the negative control, the hormone concentration after 56 days from the administration was higher than that after 28 days. On the contrary, FSH and LH levels of *Cordia*-treated groups significantly (*p* ≤ 0.05) elevated during the recovery stage compared to the negative control levels. The highest dose of *Cordia* caused the highest elevation in FSH and LH level (5.33 ± 0.17 and 2.76 ± 0.24 mIU/L, respectively), in comparison to the negative control (3.51 ± 0.30 and 2.00 ± 0.20 mIU/L, respectively).

After administering low and high doses of *Cordia* for 28-days, TS concentration did not significantly change compared to the negative control. After administration of the extract for 56 days, TS of all *Cordia* treated groups significantly decreased compared to the negative control. In the recovery stage, TS levels of all *Cordia*-treated groups were significantly elevated. The highest TS level was recorded with the group treated with the highest dose of *Cordia* (500 mg/kg); 4.05 ± 0.18 nmol/L, compared to TS of negative control (3.12 ± 0.26 nmol/L) at the same time.

#### The effect on seminal quality

*Cordia* treatment had a significant effect on male fertility. *Cordia* showed a negative impact on the seminal quality of treated rats compared to the negative control. Furthermore, almost *Cordia* recorded a significant (*p* ≤ 0.05) reduction of sperm concentration, sperm motility, and alive sperm% concurrent with a substantial elevation of abnormality% in the treatment stage that considered antifertility effect. Fortunately, rats overcame the harmful impact of *Cordia* on fertility during the recovery stage (Table 4).

Sperm concentration of *Cordia*-treated rats was significantly reduced, except the low dose group, compared to the concentration of negative control after administration for 28 days (*p* ≤ 0.05). Medium and high doses dramatically declined sperm production that reached 64.00 ± 1.33 × 10^6^ and 52.04 ± 3.64 × 10^6^ sperm/mL, respectively, with 8.35% and 25.48% reduction less than the negative control. After 56 days, *Cordia* at 250 and 500 mg/kg dramatically decreased sperm concentration (61.50 ± 3.62 × 10^6^ and 47.60 ± 2.27 × 10^6^ sperm/mL with 15.08 and 34.27 reduction percentage), compared to the negative control (72.42 ± 5.75 × 10^6^ sperm/mL). Whereas, force-fed low dose significantly (*p* ≤ 0.05) raised sperm concentration to 79.50 ± 5.67 × 10^6^ sperm/mL in comparison to the negative control (72.42 ± 5.75 × 10^6^ sperm/mL). In the recovery stage, reversible effect was observed, where the highest dose of *Cordia* recorded the highest sperm concentration (76.20 ± 4.64 × 10^6^ sperm/mL) with a 15.45% increase compared to the negative control (66.00 ± 6.93 × 10^6^ sperm/mL) (Table 4).

Concerning the antifertility effect of *Cordia* treatment, it significantly reduced (*p* ≤ 0.05) sperm motility compared to the negative control. Furthermore, administration of *Cordia* for either 28 or 56 days significantly decreased sperm motility compared to the negative control. The weakest sperms occurred in the 500 mg/kg group during the treatment stage. On the contrary, sperm of 500 mg/kg group during the recovery stage became the fastest, compared to the motility of negative control; 65.00 ± 1.29% and 52.00 ± 2.49%, respectively.

Fortunately, administration of *Cordia* for 28 days significantly (*p* ≤ 0.05) raised alive sperm percentage, except for high dose, compared to the negative control percentage. Low and medium doses of *Cordia* significantly protected sperms and remained alive more than in the negative control; 84.76 ± 1.49, 78.33 ± 1.81, and 72.00 ± 2.37%, respectively. Continuing administration of *Cordia* for 56 days significantly decreased alive sperm than that of the negative control, except low dose that caused a non-significant decrease. *Cordia*-treated animals showed a reversible effect in the recovery stage that represented a significant elevation in alive sperm%, compared to the negative control.

*Cordia* significantly increased sperms abnormality percentage compared to the negative control in the treatment and recovery stage. The abnormality percentage increased parallel to *Cordia* doses increased. A high dose of *Cordia* only significantly (*p* ≤ 0.05) decreased sperm abnormality percentage lesser than negative control in the recovery stage; 17.33 ± 0.15 and 19.60 ± 0.50%, respectively.

#### Effect on the relative weight of reproductive organs

Compared to the negative control, the relative weight of reproductive organs was significantly (*p* ≤ 0.05) affected with *Cordia* administration (Table 5). *Cordia* high dose after 28 days significantly increased the relative weight of testes compared to the negative control value (*p* ≤ 0.05). However, after day 56 and day 90, *Cordia* did not cause a significant change in the relative weight of tests compared to the negative control.

**Table 5.** The relative weight of reproductive organs of male rats treated with *Cordia dichotoma* fruits ethanolic extract compared to the negative control.

	Parameter			
Groups	Relative weight of organs			
	Testes	Epididymis	Prostate	Seminal vesicles
After administration for 28-day				
Negative control	1.04 ± 0.07^bc^	0.337 ± 0.07^c^	0.192 ± 0.04^b^	0.313 ± 0.02^de^
Cordia 125 mg/kg	1.04 ± 0.15^bc^	0.340 ± 0.05^c^	0.141 ± 0.02^bc^	0.324 ± 0.05^de^
Cordia 250 mg/kg	1.02 ± 0.05^bc^	0.345 ± 0.03^c^	0.135 ± 0.01^bc^	0.280 ± 0.04^e^
Cordia 500 mg/kg	1.27 ± 0.07^a^	0.332 ± 0.02^c^	0.150 ± 0.01^bc^	0.277 ± 0.03^e^
After administration for 56-day				
Negative control	1.07 ± 0.08^bc^	0.370 ± 0.02^c^	0.171 ± 0.01^b^	0.384 ± 0.02^cde^
Cordia 125 mg/kg	0.99 ± 0.09^d^	0.350 ± 0.04^c^	0.147 ± 0.03^bc^	0.348 ± 0.01^de^
Cordia 250 mg/kg	1.02 ± 0.08^bc^	0.381 ± 0.04^c^	0.192 ± 0.01^b^	0.415 ± 0.04^bcd^
Cordia 500 mg/kg	1.04 ± 0.08^bc^	0.463 ± 0.05^b^	0.249 ± 0.03^a^	0.583 ± 0.02^a^
Recovery stage				
Negative control	1.16 ± 0.11^ab^	0.533 ± 0.02^a^	0.160 ± 0.01^b^	0.427 ± 0.04^abcd^
Cordia 125 mg/kg	1.13 ± 0.09^bc^	0.467 ± 0.03^b^	0.170 ± 0.02^b^	0.512 ± 0.02^ab^
Cordia 250 mg/kg	1.16 ± 0.15^ab^	0.463 ± 0.03^b^	0.173 ± 0.01^b^	0.473 ± 0.02^abc^
Cordia 500 mg/kg	1.13 ± 0.09^bc^	0.460 ± 0.06^b^	0.181 ± 0.07^b^	0.478 ± 0.02^abc^

Data presented as mean ± SE. Data were analysed using ANOVA one-way followed with *post hoc* for multiple comparisons and *P < 0.05*. The means followed by the same letter in each column are not significantly different from each other at the 5% probability level (Duncan’s multiple range test).

Relative weight of organ = (absolute weight of organ/ total body weight) × 100.

After 28 days, epididymis and prostate relative weight did not significantly change compared to the negative control. Meanwhile, after day 56, epididymis and prostate relative weight were raised considerably in groups treated with high doses of *Cordia* (0.463% ± 0.05 and 0.249% ± 0.03, respectively), compared to the negative control (0.370 ± 0.02% and 0.171 ± 0.01%, respectively). Finally, at the recovery stage, the relative weight of epididymis of *Cordia*-animals significantly decreased. Meanwhile, the relative weight prostate was increased considerably compared to the negative control animals.

*Cordia* did not significantly change the relative weight of the seminal vesicle after 28-days. However, after 56 days, a high dose significantly increased the relative weight of the seminal vesicle, compared to the negative control; 0.583 ± 0.02 and 0.384 ± 0.02%, respectively (*p* ≤ 0.05). Thus, at the recovery stage, the relative weight of the seminal vesicle of treated animals was close to the relative weight of the negative control.

#### The effect on histology of the reproductive organs

**Figure 3.**
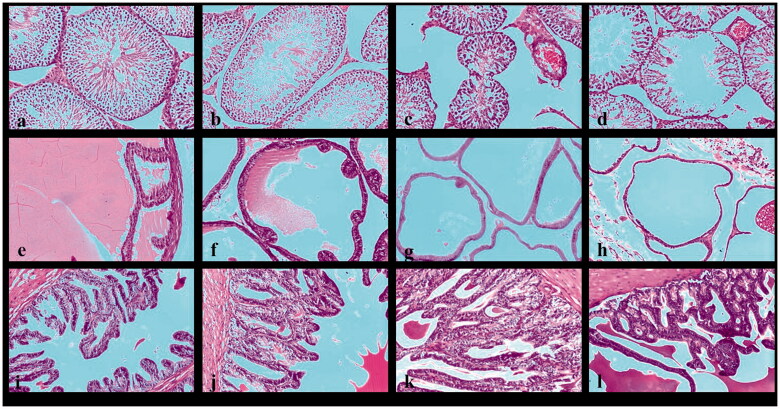
Photomicrographs of 28-days. The negative control testis section of the negative control group shows typical oval to rounded seminiferous tubules (a). Photomicrograph of testis section of the 125- and 250 mg/kg-treated groups showing mild atrophy of seminiferous tubules associated with mild interstitial edema and congested blood vessels (b,c). Testis section of the 500 mg/kg-treated group showed mild atrophy of seminiferous tubules concurrent with mild degenerated and pyknotic spermatogonial cells. Notice the absence of spermatid cells (d). Prostate section of the negative control group showing normal prostatic acini with intraluminal prostatic secretions (e). Prostate section of 125 mg/kg-treated group showing mild atrophy of prostatic acini associated with mild degeneration of lining epithelium (f). Prostate section of 250 mg/kg-treated group showing mild atrophy of the prostatic acini related with mild peri acinar fibrosis and mild degeneration of lining epithelium (g). Prostate section of 500 mg/kg-treated group showing the prostate, acinus – atrophy associated with mild degeneration of lining epithelium and absence of secretions (h). Photomicrograph of the seminal vesicle section of the negative control group shows a typical gland's typical histologic structure (I). Seminal vesicle section of 125, 250, and 500 mg/kg-treated groups showed mild hyperplasia of the lining epithelium (j–l) (H & E staining × 100).

The histopathological examination of the testicular sections of the negative control rats showed typical histological architecture of seminiferous tubules, spermatogonial cells, Leydig cells, Sertoli cells, and blood vessels (Figure 3(a)). Examined sections in 125 mg/kg-treated group receiving 125 mg *Cordia* for 28-day showed mild atrophy of seminiferous tubules associated with mild interstitial edema (Figure 3(b)).

Administration of 250 mg *Cordia* for 28-day to the rats in the 250 mg/kg-treated group showed testicular sections with mild atrophy of seminiferous tubules, mild interstitial edema, and congestion of blood vessels within the testis (Figure 3(c)). However, the 500 mg/kg-treated group's examined section showed severe atrophy of seminiferous tubules associated with mild degenerated and pyknotic spermatogonial cells (Figure 3(d)).

Histopathologic examination of the prostate sections obtained from the rats in the negative control group showed normal prostatic acini with typical glandular epithelium structure of prostate glands associated with the presence of intraluminal prostatic secretions (Figure 3(e)), the prostatic histopathological examination of 125 mg/kg-treated group showed mild atrophy of prostatic acini associated with mild degeneration of lining epithelium (Figure 3(f)), while 250 mg/kg-treated group examined sections showed mild atrophy of the prostatic acini related with mild peri acinar fibrosis and mild degeneration of lining epithelium (Figure 3(g)), the prostatic histopathological examination of 500 mg/kg-treated group showed prostate, acinus-atrophy associated with mild degeneration of lining epithelium and absence of secretions (Figure 3(h)).

Histopathologic examination of the seminal vesicle sections of negative control rats showed the typical histologic structure of the gland, with columnar lining epithelium and dense fluid (Figure 3(i)). Treatment with *Cordia* in all groups showed mild hyperplasia of the lining epithelium, respectively (Figure 3(j)–(l)).

**Figure 4.**
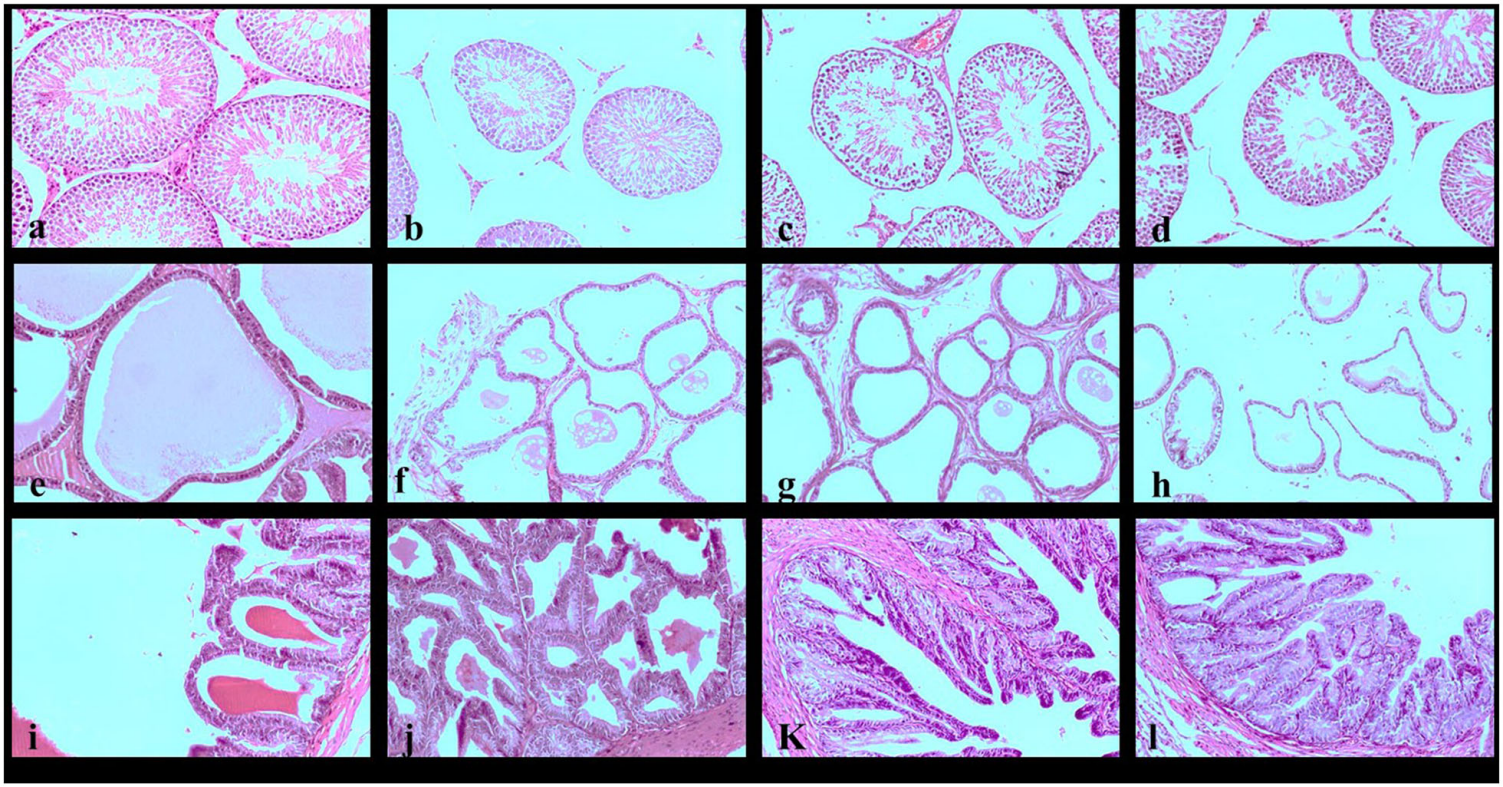
Photomicrograph of 56 days. The negative control testis section showed a typical structure of the testicular tissue (a). Photomicrograph of testis section of the 125 mg/kg-treated group showing normal seminiferous tubules associated with mild interstitial edema (b). Testis section of the 250 mg/kg-treated group showed severe atrophy of the seminiferous tubules related to extreme degenerative changes, interstitial edema, and congested blood vessels (c). Testis section of the 500 mg/kg-treated group showed severe atrophy of seminiferous tubules associated with extreme degenerative changes, interstitial edema, and congested blood vessels. The absence of spermatid cells was noticed (d). Prostate section of the control and 125 mg/kg-treated groups showing normal prostatic acini with intraluminal prostatic secretions (e,f). Prostate section of 250 mg/kg-treated group showing mild atrophy of prostatic acini associated with mild degeneration of lining epithelium (g). Prostate section of 500 mg/kg-treated group showing the prostate, acinus - atrophy associated with mild degenerating lining epithelium and absence of secretions (h). Photomicrograph of the seminal vesicle section of the control group showed a typical histologic structure of the gland (i). Photomicrograph of seminal vesicle sections of 125, 250, and 500 mg/kg-treated groups showed mild hyperplasia of the lining epithelium (j, k, and l) (H & E staining × 100).

The severity of the pathologic lesions is increased with the doses administered and increased the time by 56 days. Testes of negative control rats showed typical oval to rounded seminiferous tubules with normal lining epithelium (Figure 4(a)). The 125 mg/kg-treated group showed normal seminiferous tubules associated with mild interstitial edema (Figure 4(b)). Examined sections of the 250 mg/kg-treated group showed severe tubular atrophy of seminiferous tubules appearing irregular in shape associated with extreme degenerative changes of the lining spermatogonial cells, interstitial edema, and congested blood vessels (Figure 4(c)). 500 mg/kg-treated group, on the other hand, showed severe atrophy of seminiferous tubules associated with extreme degenerative changes, interstitial edema, congested blood vessels, severe degeneration of Sertoli cells, and marked degeneration of Leydig cells (Figure 4(d)).

Histopathological examination of negative control and 125 mg/kg treated group prostate sections showed normal prostatic acini with intraluminal prostatic secretions (Figure 4(e,f)). The 250 mg/kg treated group examined sections showed mild atrophy of prostatic acini associated with mild degeneration of lining epithelium (Figure 4(g)). The prostate of 500 mg/kg treated group, on the other hand, showed prostate, Acinus–Atrophy, characterised by decreased acinar size, atrophy of the lining epithelial cells, the scanty or total absence of the secretory material (Figure 4(h)).

Examined sections of the seminal vesicle of the negative control group showed the typical histologic structure of the gland (Figure 4(i)). In all groups, treatment with *Cordia* (125, 250, and 500 mg/kg) showed mild hyperplasia of the lining epithelium, respectively (Figure 4(j–l)).

**Figure 5.**
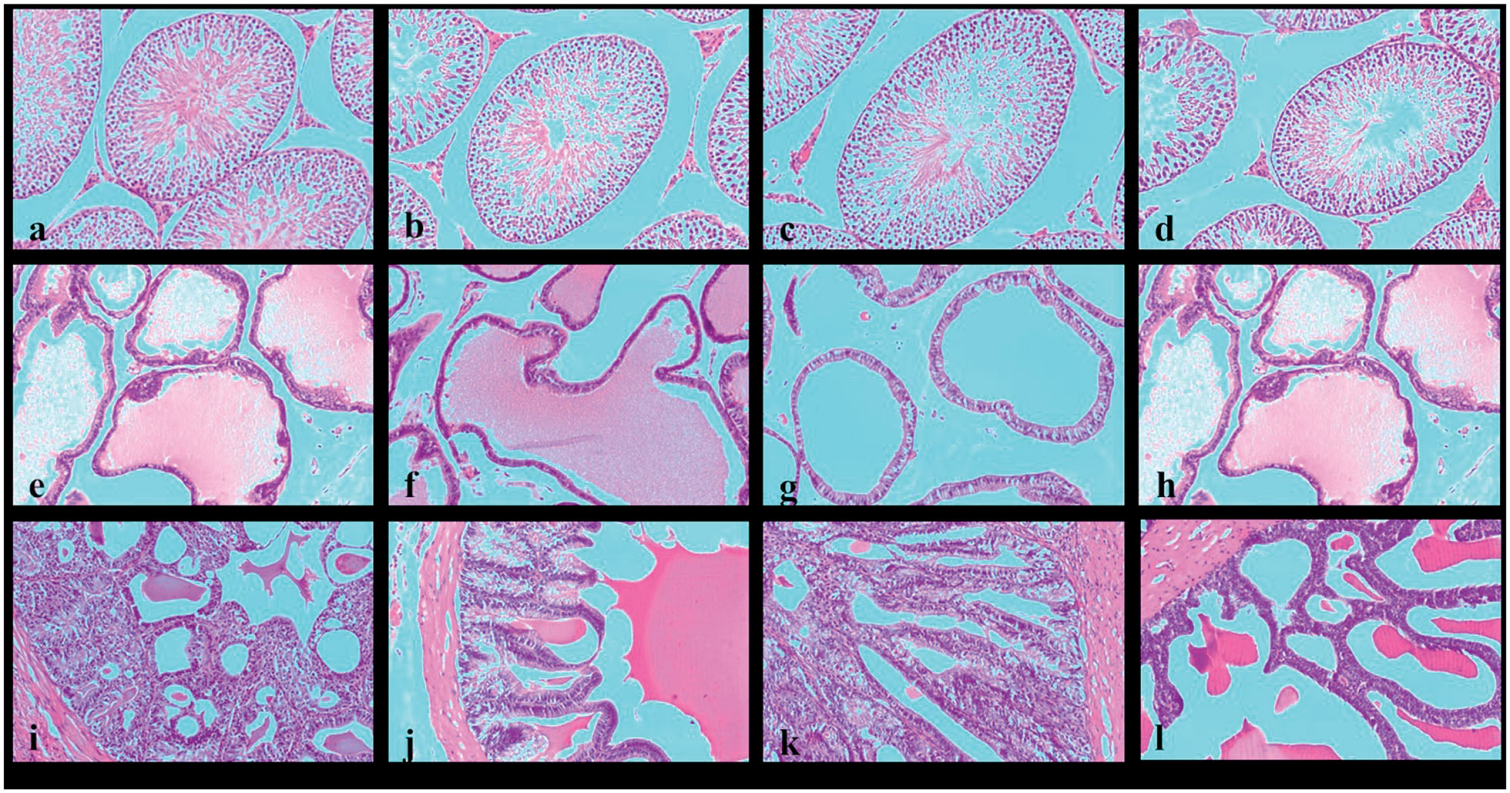
Photomicrograph of 90-days. Photomicrograph of testis section of the negative control group showing a typical oval to rounded seminiferous tubules (a). Testis section of the 125, 250, and 500 mg/kg-treated groups showed normal seminiferous tubules associated with mild interstitial edema (b–d). Photomicrograph of the prostate section of the negative control group showing normal prostatic acini with intraluminal prostatic secretions (e). Photomicrograph of the prostate section of 125, 250, and 500 mg/kg-treated groups showed normal prostatic acini with intraluminal prostatic secretions (f–h). Photomicrograph of seminal vesicle section of the negative control group showing a typical histologic and hyperplastic structure of the gland (i). Seminal vesicle section of 125, 250, and 500 mg/kg-treated groups showed mild hyperplasia of the lining epithelium (j–l) (H & E staining × 100).

Animals at the recovery stage showed a significant degree of recovery in all the examined organs (Figure 5(a)), and all the *Cordia*-treated (125, 250, and 500 mg/kg) showed normal seminiferous tubules associated with mild interstitial edema (Figure 5(b)–(d)).

The prostate of negative control rats showed normal prostatic acini with the presence of intraluminal prostatic secretions (Figure 5(e)). The same improvement was detected in all the *Cordia*-treated groups, where prostate examined sections showed normal prostatic acini, with normal lining epithelial cells and the presence of secretory materials (Figure 5(f–h)).

Examined sections of seminal vesicle of the negative control and all *Cordia*-treated groups showed the glandular lumen's typical histologic and hyperplastic structure characterised by newly formed papillary structures projecting into the glandular lumen (Figure 5(i–l)).

## Discussion

The current study revealed that administration of *Cordia* extract at 125, 250, and 500 mg/kg/day for 56 days caused a significant antifertility effect on male rats. Furthermore, antifertility effects were shown as a considerable deterioration in sperm production, motility, normality, and alive percentage of sperm and a significant drop of FSH, LH, and TS synthesis, particularly with high doses. Fortunately, the animals could remove this antifertility effect after one month from stopping *Cordia* administration while retaining the good effects. Therefore, the antifertility effect of *Cordia* was considered a revisable effect.

These results are supported by histopathological examination. Where rats fed on *Cordia dichotoma* showed mild to severe atrophy of seminiferous tubules and prostate cells and hyperplasia of the seminal vesicle in a dose-dependent manner. These results were in accordance with Sharma et al. (2015) they concluded that the antifertility effect of *Cordia dichotoma* might be as a result of the presence of certain oestrogenic phytoconstituents in the plant as well as its potent contraceptive potential due to its strong oestrogenic potential **(**Bhattacharya and Saha 2013). It is suggested that the distortion in seminiferous tubules is due to the loss of germ cells by cell degeneration and detachment of Sertoli cells from the basal lamina resulting in the decrease in diameter due to atrophy of the tubules, massive spermatogenic necrosis (Agarwal et al. 2012). The decreased relative weight of the prostate and seminal vesicle is relevant to histopathological examination expressing atrophy and peri acinar fibrosis; the seminal vesicle and prostate weight can be used as a crude measurement of circulating testosterone levels. However, they may also reduce their weight if there is interference with testosterone binding at the surface receptor; weight reduction can also occur if bodyweight decreases (Wanda et al. 2010). These pathologic alterations were diminished when rats were kept for 30 days post-treatment showing a significant degree of recovery; the seminiferous epithelium usually recovered its reproductive capability and restored normal fertility. The increased serum and intratesticular testosterone levels are attributed to the recovered population of Leydig cells; this androgen binding with receptors located in Sertoli cells maintains the blood-testis barrier of these cells and formation of proper adhesion between Sertoli cells and spermatids as postulated by Walker (2011). Also, Leydig cells can spontaneously recover faster than other cells, restoring normal concentrations of androgens (Sagaradze et al. 2019).

From previously explained data, authors need to discuss two observations. The first one was the behaviour of FSH hormone after administration of the extract for 28 and 56 days, where FSH hormone levels of *Cordia* treated groups after administration *Cordia* 56 days were higher than those after administration *Cordia* for 28 days. These results may be due to the enhancement of the pituitary ovary axis activities, which resulted in increasing the level of FSH and oestrogen as discussed by Amudha and Rani (2020); the histological and biochemical estimations showed a reversible contraceptive potential after withdrawal, suggesting a phytopharmaceutical with potential antifertility activity with reversible safety aspects (Bhattacharya and Saha 2013). The second observation was a significant decrease in TS, which was recorded only with the medium dose of *Cordia* after administration for 28 days, where the increased oestrogen levels increased the cortisol-binding globulin and the free cortisol level elevated, oestrogen also decreased the ability of the liver to metabolise cortisol and contributed to the elevation of unbound cortisol (Speroff et al. 1999), resulting in reducing the levels of testosterone, due to binding with serum proteins.

The hypothalamic-pituitary-gonadal axis controls the spermatogenesis process. The hypothalamus gland secretes gonadotropin-releasing hormone (GnRH) that promotes FSH and LH hormones from pituitary glands. Leydig cells in interstitial tissue of tests produce TS under the LH effect. FSH stimulates Sertoli function. FSH and TS synergistically work to produce sperm cells in seminiferous tubules by meiotic division. Produced sperms pass through the epididymis, those secret substances for sperm maturation. Finally, seminal vesicles and prostate glands secret semen components as fructose, citrate, inositol, and prostaglandins. Success spermatogenesis process is dependent on the harmony between the previous steps. Therefore, any pathological alternation in the male reproductive system may interfere with fertility via impairment of TS level or disturbing spermatogenesis and sperm maturation (Cooper 2002; Hafez and Hafez 2005).

Cholesterol (TC) is vital for the male reproductive system. TC is the precursor of steroid synthesis, the major TS source, crucial for normal spermatogenesis. The steroidogenesis process is responsible for TS production from TC via several steps as *de novo* synthesis. Therefore, depletion in TC due to genetic modification in the mouse model led to infertility (Parton and Hancock 2004). The main source of steroid synthesis in plasma lipoproteins. Also, Leydig cells can produce TC through the *de novo* process and use stored TC (Stocco et al. 2005).

Indeed, sperm production is strongly associated with TC on the excellent mass production of germ cells during spermatogenesis. Therefore, TC *de novo* synthesis was elevated during the development of pachytene, leptotene, and zygotene stages to increase germ cells' diameter and surface area (Sèdes et al. 2018). Then TC synthesis decreased, and TC hydrolysed to cholesterol ester by the hormone-sensitive lipase (HSL), which involves in spermatids and spermatozoa elongation. Therefore, any disruption in lipid metabolism during sperm production can lead to arrest cell differentiation. Spermatozoa, like any animal cell, have a lipid bilayer plasma membrane. Polyunsaturated fatty acids (PUFAs) make spermatozoon more viable and dynamic (Sèdes et al. 2018). Germ cells produce TC by de novo and supply with TC in the seminiferous tubules to increase their membrane surface (Akpovi et al. 2006).

Additionally, lipids strongly affect spermatozoa post-testicular maturation. In the epididymis, the lipid composition of spermatozoa gives it greater membrane fluidity. The epididymis also synthase lipid vesicles to facilitate protein transfer to spermatozoa. Finally, spermatozoa capacitation, the post-testicular maturation, strongly depends on the lipid composition of the spermatic membrane (Jin and Yang 2017).

From the previous explanation, we can observe the importance of the lipid profile of animals for fertility and normal embryonic development resulting insufficient sperm count, good motility percentage, acceptable alive percentage, and low sperm abnormality, which lead to successful fertility. Unfortunately, in this study, *Cordia* extract reduced TC, the precursor of the steroidogenesis process, by about 20.21 and 29.02% after administration for 28 and 56 days, respectively. Therefore, the high dose recorded the highest antifertility effect. Also, *Cordia* extract showed a hypoglycaemic effect. Glucose converts to pyruvate and lactate to provide post-mitotic germ cells with energy reduced after administration for 28 days, 56 days, and recovery stages.

Finally, we can conclude that the antifertility effect that appeared by *Cordia* fruits may be attributed to the reduction of FSH and LH that reflected TS production and seminal quality. Besides the hypolipidemic and hypoglycaemic effects of *Cordia* that reduced cholesterol, the steroid cycle's precursor.

## Conclusions

*Cordia* harmed male fertility during two months of dosing. A significant deficit in sperm count, motility%, alive%, concurrent with a substantial increase in abnormalities%, were recorded. Also, it significantly reduced FSH, LH, and testosterone levels. In addition, a negative alternation in reproductive organs, including tests, prostate, and seminal vesicles. Fortunately, animals can restore their normal fertility within one month from stopping *Cordia* administration. The antifertility effect of *Cordia* may be attributed to its hypolipidemic effect. Where *Cordia* significantly minified the cholesterol concentration of treated rats. Therefore, *Cordia* is considered safe for animals because it remains in the field for two months annually. If the animals eat *Cordia* within two months, then they can restore their fertility within one month.
